# The Effect of Parental Phubbing on Depression in Chinese Junior High School Students: The Mediating Roles of Basic Psychological Needs Satisfaction and Self-Esteem

**DOI:** 10.3389/fpsyg.2022.868354

**Published:** 2022-03-29

**Authors:** Xiaofang Xiao, Xifu Zheng

**Affiliations:** ^1^Key Laboratory of Brain, Cognition, and Education Sciences, South China Normal University, Ministry of Education, Guangzhou, China; ^2^Guangdong Key Laboratory of Mental Health and Cognitive Science, School of Psychology, Center for Studies of Psychological Application, South China Normal University, Guangzhou, China

**Keywords:** junior high school students, parental phubbing, basic psychological needs satisfaction, self-esteem, depression

## Abstract

**Objective:**

To reveal the relationship between parental phubbing, basic psychological needs satisfaction, self-esteem, and depression and to explore the impact of parental phubbing on depression.

**Methods:**

A total of 819 junior high school students responded to the parental phubbing scale, basic psychological needs satisfaction scale, self-esteem scale, and depression scale in combination.

**Results:**

(1) Parental phubbing was significantly correlated with satisfaction of basic psychological needs, self-esteem, and depression. (2) Parental phubbing can not only be used to directly predict depression in junior middle school students but also has an indirect impact on depression through three pathways: a separate mediating effect on basic psychological needs satisfaction, a separate mediating effect on self-esteem and a chain mediating effect on both.

**Conclusion:**

Parental phubbing is a risk factor for depression, which can negatively affect the mental health of junior high school students.

## Introduction

With the increasing availability of the functions of smartphones, use by people in daily life and work shows an overall upward trend. As a result, the use of mobile phones consumes a great deal of time for individuals and has a considerable impact on relationships (Burchell, 2015). Of the 11 countries surveyed, nearly all adults have at least one mobile phone (Silver et al., 2019). In China, the proportion of mobile phone users reached 99.6% in 2021 (CNNIC, 2021). A common phenomenon that attracts attention from researchers is the use of mobile phones for an activity known as “phubbing”. Composed of “phone” and “snubbing,” the word “phubbing” has now been included in the latest version of the Macquarie Dictionary (Karadağ et al., 2016). This word is relatively new, referring to the behavior of engaging with a phone while ignoring others during interaction (Angeluci, 2016). When phubbing behaviors occur in the context of parent–child communication, it is defined as parental phubbing, referring mainly to the behavior of parents neglecting their children (Jiang et al., 2021). Spending excessive energy and time on mobile phones, they tend to neglect their children’s feelings (Jiang et al., 2021). Numerous studies have demonstrated that parental phubbing behaviors have a detrimental effect on the parent–child relationship (Xie et al., 2019; Niu et al., 2020; Pancani et al., 2020; Liu K. et al., 2021a,b), reducing the quality of parenting (Qu et al., 2020) and possibly resulting in teenagers developing negative emotions such as depression (Liu J. et al., 2021), loneliness (Geng et al., 2021), and internalizing problems (Wang et al., 2020). Apart from that, adolescents may be at an increased risk of internet addiction and mobile phone use as a result of parental phubbing (Hong et al., 2019). However, the use of mobile smart phones to deal with various trivial affairs has now been made commonplace and leads to parents spending less time caring for their children (McDaniel, 2019). Therefore, conducting research on the negative impact of parental phubbing is certainly conducive to understanding how this behavior exerts influence on our domestic lives and the development of children.

As a universal phenomenon among teenage groups (Jane Costello et al., 2006; Vogel, 2012), depression impedes the physical and mental development of adolescents, thus drawing much attention from all aspects of society. Due to the unstable emotions of adolescents, they are at a significantly increased risk of depression if exposed to phubbing during the puerperal period, which is a critical stage of life growth (Avenevoli et al., 2008). According to empirical studies, early depression in adolescents increases the risk of depression and anxiety disorders for them when they become adults (Fergusson and Woodward, 2002) and represents a risk factor for suicide in children and adolescents (Carballo et al., 2020). As revealed by a 10-year survey conducted in the United States, the prevalence of adolescent depression surged from 8.1% to 15.8% by 2021 (Daly, 2022). In China, screening for depression was made an essential part of health examinations for secondary school students in 2021 (Chinese Ministry of Education, 2021). In summary, depression should not go unaddressed. Thus, this study on the influence mechanism of parental phubbing on adolescents with depression could potentially lead to a reduction in depression for adolescents by providing a theoretical basis for intervention research.

### Parental Phubbing and Depression Among Adolescents

In prior studies, phubbing was routinely regarded as the act of ignoring others’ feelings or excluding others during times of potential interaction (David and Roberts, 2017). As concluded by substitution theory in the phubbing phenomenon, parents may pay attention to mobile phones rather than parent–child interactions, ignoring the feelings of their children, which is detrimental to the relationship and contributes to the negative feelings that children may already have (Al-Saggaf and O’Donnell, 2019; Jiang et al., 2021). In addition, it is believed by acceptance-rejection theory (Rohner, 2004) that a warm parenting dimension is constituted by the parental acceptance and rejection of children. One end of this dimension is acceptance, which means that children feel the acceptance and inclusion of parents, thus promoting their healthy growth. On the other end is rejection, which means children feel the neglect and exclusion of parents, thus damaging their physical and mental health. Additionally, parental phubbing is possibly a sign of rejection (Allred, 2020). According to empirical studies, parental phubbing can result in loneliness (Wang et al., 2021) and depression (Stockdale et al., 2018; Bai et al., 2020; Wang et al., 2020, 2022; Xie and Xie, 2020; McDaniel, 2021; Xie et al., 2021). In summary, this study proposes H1: Parental phubbing leads to adolescent depression.

### The Mediating Role of Basic Psychological Needs Satisfaction

Proposed by self-determination theory, competency needs, relationship needs, and autonomy needs represent three significant sections of basic human psychological needs satisfaction (Deci and Ryan, 2000). With the satisfaction of basic psychological needs, individuals feel motivated and pleased (Klein, 2019). Conversely, when basic psychological needs are not satisfied, a range of problems can occur and can negatively affect the physical and mental health of children (Klein, 2019). Basic psychological needs satisfaction acted as an essential mediator of adolescents’ psychological and behavioral development within the environmental impacts of school and family (Wu et al., 2018). According to social exclusion theory, human beings have a basic original motivator of avoiding being excluded by social groups, which means that being a social member could increase individual existence probability (Baumeister and Tice, 1988). In contrast, being excluded results in negative feelings which can lead to such issues as depression, anxiety and loneliness (Leary, 1990). Parental phubbing is considered a new kind of neglect and exclusion behavior (Roberts and David, 2016; David and Roberts, 2017). Based on previous studies, parental phubbing was regarded as neglect and exclusion behavior, which can harm the parent–child relationship and impact the mental health development of children (Xie and Xie, 2020; Wang et al., 2021). Exclusion and neglect in interaction may have a passive impact on the mental health of the people who are excluded and may lead to them not getting their basic psychological needs met (Yang, 2020). Therefore, parental phubbing might leave adolescents’ basic psychological needs unsatisfied. From the viewpoint of self-determination theory, human beings’ basic psychological needs can be satisfied or harmed in social surroundings (Deci and Ryan, 2008). Basic psychological needs satisfaction is considered as a protective factor against depression (Wei et al., 2005; Chang et al., 2015). In addition, some studies explained depression *via* basic psychological needs satisfaction (Véronneau et al., 2005; Emery et al., 2015). Emery et al. (2015) found that depression symptoms during childhood were negatively correlated with basic psychological needs satisfaction, and the satisfaction degrees of competency needs and relationship needs were also passively correlated with adolescents’ basic psychological needs satisfaction. At the same time, a longitudinal study showed that satisfaction of basic psychological needs could predict depression among teenagers (Zhong et al., 2020). These studies all found that basic psychological needs satisfaction could predict adolescents’ depression. Apart from this, empirical research has depicted that parental phubbing can lead to depression in teenagers by damaging relationship needs (Xie and Xie, 2020). Liu K. et al. (2021a) also found that parental phubbing may lead to adolescents feeling that their basic psychological needs have not been satisfied. In previous empirical studies, it has been demonstrated that basic psychological needs satisfaction can mediate the correlation between general exclusion, parental corporal punishment and adolescent aggressive behavior (Yu et al., 2018; Zhou et al., 2021). However, few studies have explored the relationship between parental phubbing and basic psychological needs satisfaction. As a result, this study proposes Hypothesis H2: basic psychological needs satisfaction plays a mediating role in parental phubbing and adolescent depression.

### The Mediating Role of Self-Esteem

In definition, self-esteem is the evaluation of individual self-worth or value (Cast and Burke, 2002). According to the sociometer theory, social exclusion causes self-esteem to diminish (Leary et al., 1995a). In other words, self-esteem as an interaction monitor, whether an individual perceives acceptance or rejection, determines the high and low levels of self-esteem (Leary et al., 1995b). The parent–child relationship is regarded as the most important social relationship in an individual’s life and is closely correlated with children’s self-esteem (Hong et al., 2019). Empirical studies have illustrated that better parental attachment and family cohesion boosted self-esteem (Wilkinson, 2004; Lian and Yusooff, 2009), while parental phubbing could diminish adolescents’ self-esteem (Hong et al., 2019). From another perspective, a model of susceptibility to depression posits that low self-esteem contributes significantly to adolescent depression (Orth et al., 2008; Orth and Robins, 2013). A previous longitudinal study showed that low self-esteem is an important predictor of depression (Sowislo and Orth, 2013; Zhou et al., 2020). Some cross-sectional studies have also demonstrated that low self-esteem is a significant predictor of depression (Fiorilli et al., 2019). Therefore, self-esteem plays a critical role in the development of adolescent mental health as an essential intermediary variable (Yang et al., 2004). Self-esteem has the potential to mediate the relationship between parental phubbing and adolescent depression. Some studies have revealed that self-esteem can play a significant mediating role not only between parental rejection and depression but also between social exclusion and depression (Lei et al., 2018; Peng et al., 2021). Moreover, empirical studies have been conducted to suggest that parental phubbing reduces the core self-evaluation of adolescents (Liu K. et al., 2021a). Therefore, this study proposes Hypothesis H3: Self-esteem plays a mediating role between parental phubbing and depression.

### The Chain Mediating Effect of Basic Psychological Needs Satisfaction and Self-Esteem

From the viewpoint of self-determination theory, basic psychological satisfaction is regarded as one of the most important fountain sources of the formulation and development of self-esteem (Ümmet, 2015). The hierarchy of needs theory closely connects individual self-esteem with basic psychological needs satisfaction (Fan et al., 2020), which suggests that self-esteem cannot be enhanced until basic psychological needs are satisfied. Ümmet (2015) illustrated that when college students’ psychological needs are satisfied, their self-esteem may increase. There are numerous prior studies confirming that basic psychological needs satisfaction are closely associated with self-esteem. According to an empirical study of middle school students, basic psychological needs satisfaction can be used to predict self-esteem (Demirtas et al., 2017). In other empirical studies, it has also been demonstrated that satisfaction of basic psychological needs and self-esteem play a chain mediating role between social support and depression (Moradi and Cheraghi, 2015), and the higher the satisfaction of basic psychological needs, the higher the self-esteem (Butkovic et al., 2020). Additionally, an empirical study was carried out in China to reveal that basic psychological needs satisfaction and self-esteem play a chain mediating role between emotional abuse and social anxiety among children (Bu et al., 2017). On this basis, this study proposes Hypothesis H4: basic psychological needs satisfaction and self-esteem play a chain mediating role between parental phubbing and depression.

### Present Study

Currently, many studies have revealed that phubbing contributes to depression in adolescents, despite a lack of empirical research that investigates the impact mechanism of phubbing on adolescent depression. This study is aimed not only at revealing the correlation between parental phubbing and depression but also at explaining the mediating effect of basic psychological needs satisfaction and self-esteem. Based on the review of the relevant studies, we had the following tentative hypotheses:

*Hypothesis 1:* Parental phubbing leads to adolescent depression.

*Hypothesis 2:* Basic psychological needs satisfaction plays a mediating role between parental phubbing and adolescent depression.

*Hypothesis 3:* Self-esteem plays a mediating role between parental phubbing and depression.

*Hypothesis 4:* Basic psychological needs satisfaction and self-esteem play a chain mediating role between parental phubbing and depression.

**Figure 1:**
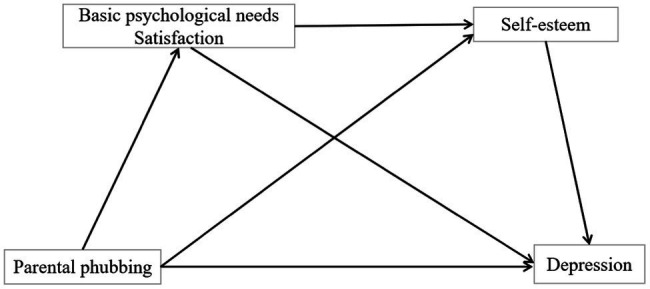
Relationship path map of parental phubbing, basic mental adequacy, self-esteem and depression.

The relationship path diagram proposed in this study is illustrated in Figure 1 as follows.

## Materials and Methods

In the next section, a discussion will be conducted about the sampling strategy, participant information, questionnaire information (i.e., item numbers, response scales, reliability, and validity), and the methods of data analysis.

### Participants and Procedure

A convenience sampling method was adopted to select three junior middle schools in Guigang city of Guangxi Zhuang Autonomous Region from grade 1 to grade 3. The current study was approved by the research ethics board of the University. The class was taken as a unit to carry out group testing, and a total of 819 valid anonymous paper questionnaires were collected. Among the junior middle school students participating in the survey, 563 were from rural areas, 256 were from urban areas, 379 were male, 440 were female, 268 were junior one, 284 were junior two, and 267 were junior three. Their ages ranged from 11 to 18, the average age was 13.48, and the standard deviation was 0.98.

### Instruments

#### Parental Phubbing

Revised by Ding et al. (2020), the parental phubbing scale was applied. As a single dimension, the scale involved 9 items in total (Roberts and David, 2016). Aside from the reverse score of the seventh item, it was a positive score. Given the Likert-5 score ranging from “never so” to “always so,” the higher the total score, the more severe the partner phubbing. In this study, the Cronbach’s *α* value of the questionnaire was 0.72.

#### Basic Psychological Needs Satisfaction

The basic psychological needs satisfaction Scale (BPNS) was developed using Gagné (2003) and revised in Chinese with good validity among adolescents (Liu et al., 2013). It consists of 19 items categorized into three dimensions: competency needs, autonomy needs, and attribution needs (Johnston and Finney, 2010). The five-point scoring method is applied, where nine items are scored in reverse. The scale’s total score is used to measure the level of basic psychological needs satisfaction, and the higher total the score is, the higher the degree to which the basic psychological needs satisfaction is met. Previous study showed that the scale had a good reliability and validity when used with Chinese adolescents (Sun et al., 2020). In this study, the Cronbach’s α value of the questionnaire was 0.75.

#### Self-Esteem

The Self-esteem scale revised by Rosenberg (1965) was adopted with the original scale involving 10 questions in total. It can be used in Chinese context with good reliability and validity (Luo et al., 2020). This study removed the eighth question from the original scale as it is not applicable to Chinese culture (Shen and Cai, 2008), thus, only nine questions were raised. The five-point scoring method is applied, where 3, 5, 8, and 9 are reverse scoring, the scores of which are positive scoring. Then, the scores of the nine questions are added. The higher the total score, the higher the level of self-esteem. In this study, the Cronbach’s α value of the questionnaire was 0.77. Confirmatory factor analysis was conducted.

#### Depression

Revised by He et al. (2013), a simplified version of the Center for Epidemiological Depression Scale was adopted. The scale consisted of nine questions with two dimensions. The third and fifth questions were reverse scored, and the score was positive. The higher the score is, the greater the degree of depression (Radloff, 1977). In this study, the Cronbach’s α value of the questionnaire was 0.76.

### Common Method Biases

The Harman single-factor test method was applied to process all measurement items through nonrotating exploratory factor analysis. According to the analytical results, there are a total of 12 common factors with eigenvalues greater than 1 extracted, and the first common factor can be used to explain 7.94% of the total change, which falls short of the 40% standard threshold. That is, there is no deviation caused by the same method for data collection in this study (Podsakoff et al., 2003).

## Results

In the following section, the means and correlation coefficients between variables as well as the results of the mediation model and moderated mediation model are presented.

### Description Statistics

Table 1 shows the results of descriptive statistics and correlation data of the research variables. Parental phubbing shows not only a positive correlation with depression but also a negative correlation with basic psychological needs satisfaction and self-esteem. Basic psychological needs satisfaction is positively correlated with self-esteem to a significant extent and negatively correlated with depression. Self-esteem is significantly negatively correlated with depression.

**Table 1:** Descriptive statistics and correlation of variables.

	1	2	3	4
1. Parental phubbing	1			
2. Basic psychological needs satisfaction	−0.279^**^	1		
3. Self-esteem	−0.249^**^	0.691^**^	1	
4. Depression	0.322^**^	−0.559^**^	−0.558^**^	1
*M* ± SD	23.87 ± 5.72	80.53 ± 13.30	28.48 ± 6.27	18.87 ± 5.40

^*^p < 0.05;

** *p < 0.01*;

^***^p < 0.001.

### Parental Phubbing and Depression: Chain Mediating Effect Test

First, the predictive variables used in the study are standardized. The chain mediation effect test based on Model 6 is expanded in the process program developed by Hayes and Preacher (2013). Previous studies showed that the levels of self-esteem depend on genders (Bang et al., 2020) and geography (Vigna-Taglianti et al., 2019). Besides, the longitudinal study of Ho et al. (2018) outlined that girls reported a significant increase in the depression score from 2016 to 2017. Above all, the present study controls the ages, zones, grades, and genders. During data analysis, demographic factors were subjected to control to clarify the mediating role of basic psychological needs satisfaction and self-esteem between parental phubbing and depression. As shown in the regression equation (see Table 2), parental phubbing can be used to directly predict the depression of high school students (*β* = 0.16, *p*<0.001), parental phubbing can also promote the depression of junior high school students as a whole (*β* = 0.29, *p*<0.001), and parental phubbing can negatively predict basic psychological needs satisfaction (*β* = −0.25, *p*<0.001) and self-esteem (*β* = −0.06, *p*<0.001). Basic psychological needs satisfaction positively predicted self-esteem (*β* = 0.66, *p*<0.001) but negatively predicted depression (*β* = −0.28, *p*<0.001). Self-esteem can be used to predict depression in junior high school students directly and negatively (*β* = −0.03, *p*<0.001). The results of the regression equation are shown in Tables 2, 3. The bootstrap method was adopted for 5,000 sampling tests, and the 95% confidence interval of the three mediating paths was evaluated. As indicated by the results (see Table 3), the 95% confidence intervals of path 1, path 2, and path 3 were not 0, suggesting the significance of indirect effects produced by the three paths.

**Table 2:** Chain mediating analysis of parental phubbing and depression in junior middle school students.

Prognosis variate	Model 1 (depression)		Model 2 (Basic psychological needs satisfaction)		Model 3 (Self-esteem)		Model 4 (Depression)	
	*β*	*t*	*β*	*t*	*β*	*t*	*β*	*t*
Grade	0.03	0.39	0.066	−1.02	−0.07	1.49	0.016	0.30
Gender	0.30	4.48^***^	0.31	−4.65	−0.11	−2.16^*^	0.11	1.98
Age	0.09	1.70	0.008	−0.17	−0.07	−1.76	0.06	1.42
Source	0.36	5.17^***^	0.40	−5.62^***^	−0.04	−0.71	0.16	2.64^***^
Parental phubbing	0.29	8.95^***^	−0.25	−7.50^***^	−0.06^*^	−2.21^*^	0.16	5.46^***^
Basic psychological needs satisfaction					0.66^***^	24.42^***^	−0.28	−7.22^***^
Self-esteem							−0.30	−8.00^***^
*R* ^2^	0.16		0.14		0.49		0.40	
*F*	31.20^***^		26.29^***^		128.11^***^		78.79^***^	

* *p < 0.05*;

^**^p < 0.01;

*** *p < 0.001*.

**Table 3:** Mediating effect analysis of parental phubbing and depression.

	Indirect effect value	*BootSE*	95%CI	Relative mediating effect (%)
Total indirect effect	0.14	0.021	[0.096, 0.18]	48.27
Pathway 1	0.069	0.014	[0.044,0.099]	23.79
Pathway 2	0.018	0.0087	[0.007,0.035]	6.2
Pathway 3	0.049	0.011	[0.031,0.070]	16.89

**Figure 2:**
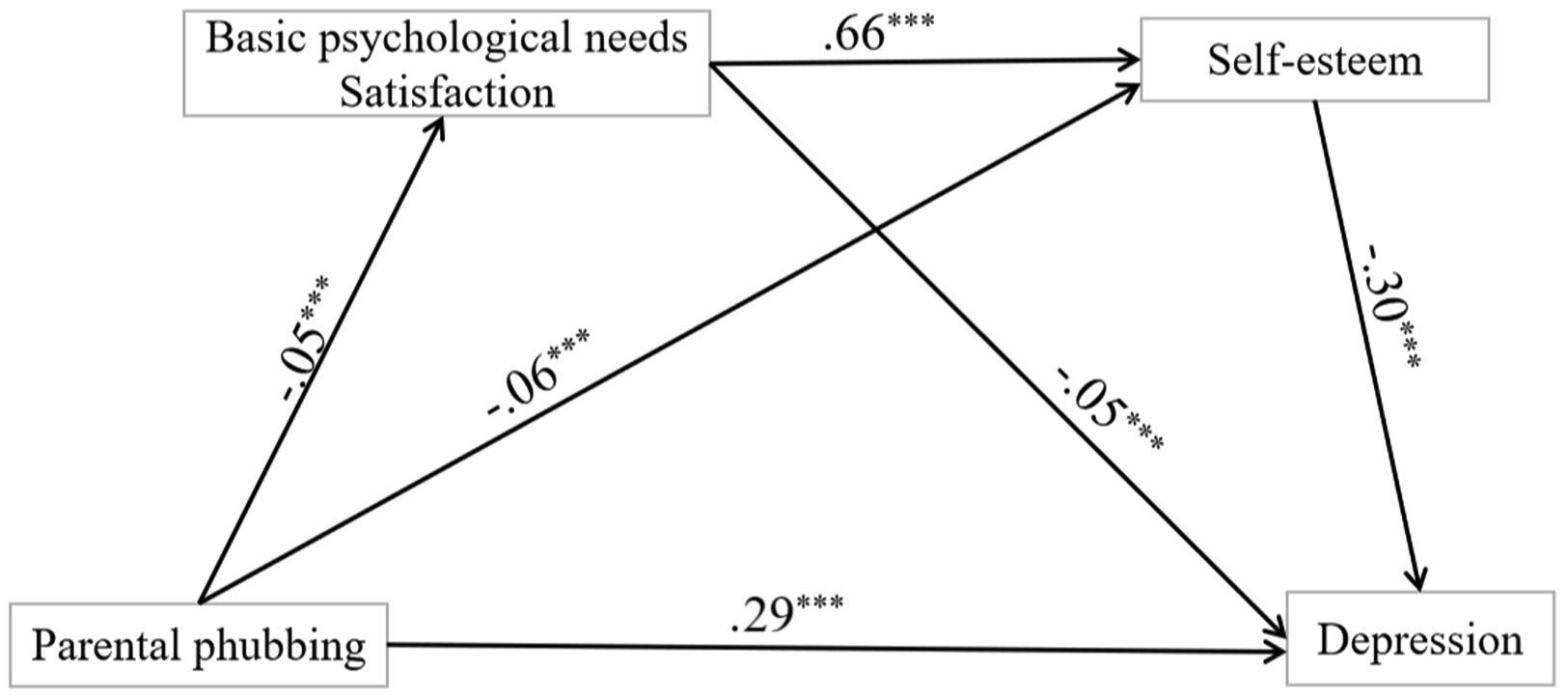
The mediating effect path map of parental phubbing and depression. *p < 0.05; **p < 0.01; and ***p < 0.001.

As revealed by the data analysis of the mediating effect (as shown in Table 3; Figure 1), basic psychological needs satisfaction and self-esteem can partially mediate the relationship between parental phubbing and depression in junior high school students. In addition, the mediating effect is 0.14, accounting for 48.27% of the total effect (0.16) that proactive social networking sites have on depression. More specifically, the indirect effects of the three pathways that formed the mediating effect are described as follows: parental phubbing→basic psychological needs satisfaction→depression was 0.069 (path 1), parental phubbing→self-esteem→depression was 0.018 (path 2), and parental phubbing→basic psychological needs satisfaction→self-esteem→depression was 0.049 (path 3). According to the data listed in Table 3, the percentages of the three indirect effects in the total effect reach 23.79%, 6.2% and 16.89%, respectively. Since 0 is not contained in the bootstrap 95% confidence intervals, these three indirect effects are self-evident (Figure 2).

## Discussion

As indicated by the results of this study, parental phubbing can contribute to depression among junior high school students, which substantiates the hypothesis proposed in this study and further confirms the negative impact that parental phubbing has on junior high school students. This is believed by substitution theory that parents spending time on mobile phones and other media devices rather than taking care of their children are unable to give children a good emotional response and interaction (Hiniker et al., 2015), thus reducing the quality of parent–child communication and undermining the parent–child relationship. Parental phubbing is regarded as a cold rejection of children (McDaniel, 2019). In some studies, it has also been revealed that parental phubbing is closely associated with parental neglect (Mun and Lee, 2021; Xie et al., 2021). According to the parental acceptance-rejection theory (Rohner, 2004), children will have depression when they feel rejected by their parents. It has also been found in previous studies that junior high school is a critical stage for the development of adolescent psychological behavior, which makes the emotional and psychological care of parents necessary, while parental neglect can lead to depression (Christ et al., 2017).

In this study, a significant negative correlation is revealed between basic psychological needs satisfaction and parental phubbing, which plays an intermediary role between parental phubbing and depression among junior middle school students, with its mediating effect accounting for 43.12%. That is, parental phubbing can contribute to depression through the damage caused to the basic psychological needs satisfaction of junior middle school students. This is consistent with the hypothesis proposed in this study. According to self-determination theory, the main basic psychological needs satisfaction that human beings have included autonomous needs, relational needs and competence needs. Any unsatisfied need will end up causing psychological or behavioral problems (Deci and Ryan, 2000). When encountering social exclusion, individuals may be hindered from the development of their mental health, which can cause negative adaptation problems due to the impairment of their relationship needs and autonomy needs (Yang, 2020). Few studies have explored the correlation between parental phubbing and adolescent mental health from the perspective of basic psychological needs satisfaction; yet some previous studies show that parental phubbing affected the relationship between adolescents and caused their life satisfaction to diminish (Xie and Xie, 2020), which provided indirect evidence for this study to some degree, as relationship needs were also one of basic psychological needs. Moreover, it is also believed by the need-threat time model that those individuals subjected to social exclusion will be destroyed and unsatisfied due to their basic psychological needs satisfaction, which can cause depression (Peng et al., 2019). As Zhou et al. (2021) suggested in empirical studies, tutor exclusion damages the mental health of graduates. In summary, phubbing is frequently defined in prior studies as a social exclusion behavior (Chotpitayasunondh and Douglas, 2018). Parental phubbing is regarded by their children as rejection, which inevitably causes depression in adolescents.

According to the results of this study, not only is self-esteem negatively linked to parental phubbing, it also plays a mediating role between parental phubbing and depression in junior high school students, with the mediating effect accounting for 11.25%, which means parental phubbing may result in depression by diminishing the self-esteem of junior high school students. This is consistent with the hypothesis proposed in this study. According to sociometer theory, exclusion in interpersonal relationships causes individual self-esteem to diminish (Leary et al., 1995b). The relationship between parents and children is essential for the development of psychological behavior in adolescents. When it is destroyed, the self-esteem of adolescents is affected. From another perspective, low self-esteem is a significant risk factor in the susceptibility model of depression (Orth et al., 2008), for which self-esteem plays an intermediary role between parental phubbing and depression. Previously, little attention was given to exploring the association between parental phubbing and the self-esteem of adolescents. In this study, it is confirmed according to social sociometer theory that parental phubbing is a risk factor for the self-esteem of adolescents. In previous studies on college students’ phubbing, it was discovered that self-esteem can produce a mediating effect between phubbing and depression (Xie et al., 2020) and that parental phubbing can also reduce the core self-evaluation of adolescents and affect their mental health (Liu K. et al., 2021a), thus providing indirect evidence for the results of this study.

As suggested by the results of this study, there is a significantly positive correlation between basic psychological needs satisfaction and self-esteem, while basic psychological needs satisfaction can be used to positively predict self-esteem. In the meantime, basic psychological needs satisfaction and self-esteem play a chain mediating role between parental phubbing and depression, with the proportion of chain mediating effect reaching 9.2%. This is consistent with the hypothesis of this study. It has also been confirmed that there is a close correlation between basic psychological needs satisfaction and self-esteem (Moradi and Cheraghi, 2015). In self-determination theory, Ümmet (2015) proved that among individuals’ basic psychological needs satisfaction, except competency needs, relationship and autonomy needs all significantly predicted self-esteem. Another study also found that the competency needs, relationship and autonomy needs within basic psychological needs all positively correlated with self-esteem, and the higher the degree of basic psychological needs satisfaction, the higher the level of self-esteem (Butkovic et al., 2020). The results of these studies were the same as those of the current study. According to Maslow’s hierarchy of needs theory, the highest level of self-realization is when self-esteem is satisfied, and only when basic psychological needs satisfaction is met can the needs for self-realization be met. When self-esteem is blocked, however, the path to self-realization may also be destroyed (Fan et al., 2020). Therefore, the healthy development of the individual body and mind depends on the critical role-played by basic psychological needs of satisfaction and self-esteem.

Depression is commonplace among adolescents and is also a risk factor for suicide and self-injury. Based on the alternative theory of phubbing, social exclusion theory and parental rejection-acceptance theory, as well as the social measurement theory of self-esteem and self-determination theory, this study explores the impact of parental phubbing on depression in junior high school students. According to the results, parental phubbing can not only cause depression among junior high school students but can also have an indirect impact on depression through three paths, with the indirect effect accounting for 36.8%. This finding reveals the significant influence of parental phubbing on the psychological development of adolescents. It not only causes adolescents to develop depression but also undermines satisfaction of their basic psychological needs and diminishes their self-esteem. Therefore, it is a risk factor for the mental health development of adolescents.

It is prompted in the research result that in future work, schools and parents can reduce teenagers’ depression starting with their self-esteem and the satisfaction of their basic psychological needs, so as to improve their subjective well-being and social adjustment. First of all, from the perspective of family education, parents should lay emphasis on the maintenance of parent–child relationship and the quality of communication, who should avoid the use of mobile phones during their communication with children as far as possible and lay emphasis on children inner demands; secondly, schools, on the one hand, can regularly host some parent–child communication activities such as parents’ meetings and parent–child coordination committees, etc., encouraging parents to put down their mobile phones and listen to their children’s mind, so as to help maintaining parent–child relationship and ensuring the quality of parent–child communication. On the other hand, educators should pay attention to the cultivation of teenagers’ self-esteem, enhancing their collective cohesion through some group guidance or collective activities, so as to provide them certain support. Finally, from the perspective of students, they should positively participate in the collective activities of schools, timely talk out to their companions or friends when feeling depressed, seek for certain help and distract their attention, meanwhile they can also express their inner demands to parents, so as to reduce their psychological pressure.

## Limitations and Future Orientation

The current research also has some deficiencies and limitations, which may be addressed in the future. First, the present study was a quantitative cross-sectional design study, not an intervention experiment, so it cannot clarify the causal relationship between variables. Therefore, future research should be conducted to better clarify the relationship between variables through both experimental design and longitudinal research. Second, for parental phubbing, research distinguishing father phubbing and mother phubbing should be carried out to explore the specific behaviors of fathers and mothers (Wang et al., 2021). The present research is not comprehensive enough to study parental phubbing overall. Future research should study father phubbing and mother phubbing to further explore the negative influence of phubbing on children. Third, implicit self-esteem still exists. This study explored explicit self-esteem (Bosson et al., 2003), but the relationship between parental phubbing and children’s implicit self-esteem remains to be further studied through experimental methods in the future.

## Conclusion

As a society with well-developed modern networks, mobile phones have become increasingly powerful. Additionally, the probability of people using mobile phones continues to rise. Given the COVID-19 pandemic, it appears that people have become increasingly reliant on their mobile phones. The high incidence of use of mobile phones by parents in the process of caring for children has a significant impact on children (McDaniel, 2019). In theory, the results of this study provide a basis for the intervention study regarding the negative impact of parental phubbing on adolescents while revealing the important role of self-esteem and basic psychological needs satisfaction.

## Data Availability Statement

The raw data supporting the conclusions of this article will be made available by the authors, without undue reservation.

## Ethics Statement

The studies involving human participants were reviewed and approved by the South China Normal University. Written informed consent to participate in this study was provided by the participants’ legal guardian/next of kin.

## Author Contributions

XX designed the research, collected the data, analyzed the data, and wrote the manuscript. XF selected the topic and worked on the final version of the manuscript. All authors contributed to the article and approved the submitted version.

## Funding

This work was supported by a grant from the National Natural Science Foundation of China (31970996), the Major Program of the National Social Science Foundation of China (19ZDA360), and psychological services and counseling bases for the Happy Guangzhou project, which received funding from the Guangzhou government.

## Conflict of Interest

The authors declare that the research was conducted in the absence of any commercial or financial relationships that could be construed as a potential conflict of interest.

## Publisher’s Note

All claims expressed in this article are solely those of the authors and do not necessarily represent those of their affiliated organizations, or those of the publisher, the editors and the reviewers. Any product that may be evaluated in this article, or claim that may be made by its manufacturer, is not guaranteed or endorsed by the publisher.
