# Supplementary material for: The Effect of Parental Phubbing on Depression in Chinese Junior High School Students: The Mediating Roles of Basic Psychological Needs Satisfaction and Self-Esteem
Source: Front Psychol. 2022 Mar 29;13:868354. doi: 10.3389/fpsyg.2022.868354 (PMC9004422; doi:10.3389/fpsyg.2022.868354)
Supplement: Supplementary file 1 [file Data_Sheet_1.doc]

**Appendix**

**1.Parental phubbing (Pphubbing) scale items**

| item | Never | Rarely | Sometimes | Somewhat frequently | Very frequently |
| --- | --- | --- | --- | --- | --- |
| 1. During a typical mealtime that my parents and I spend together, my parents pulls out and checks his/her cell phone (slight modification). | 1 | 2 | 3 | 4 | 5 |
| 2. My parents places his or her cell phone where they can see it when we are together. | 1 | 2 | 3 | 4 | 5 |
| 3. My parents keeps his or her cell phone in their hand when he or she is with me. | 1 | 2 | 3 | 4 | 5 |
| 4. When my parents's cell phone rings or beeps, he/she pulls it out even if we are in the middle of a conversation (slight modification). | 1 | 2 | 3 | 4 | 5 |
| 5. My parents glances at his/her cell phone when talking to me. | 1 | 2 | 3 | 4 | 5 |
| 6. During leisure time that my parents and I are able to spend together, my parents uses his/her cell phone (slight modification). | 1 | 2 | 3 | 4 | 5 |
| 7. My parents does not use his or her phone when we are talking. | 1 | 2 | 3 | 4 | 5 |
| 8. My parents uses his or her cell phone when we are out together. | 1 | 2 | 3 | 4 | 5 |
| 9. If there is a lull in our conversation, my parents will check his or her cell phone. | 1 | 2 | 3 | 4 | 5 |

**2.Short Depression Scale**

**Below is a list of the ways you might have fe1t or behaved. Please te11 me how often you have fe1t this way during the pasc week. HAND CARD A.During_ the past week**

| Item | Rarely or none of the Time (Less than 1 Day) | Some or a Litt1e of the Time (1-2 Days) | Occasionally or a Moderate Amount of Time (3-4 Days) | Most or A11 of the Time(5-7 Days) |
| --- | --- | --- | --- | --- |
| 1. I felt depressed . | 1 | 2 | 3 | 4 |
| 2.I fe1t that I could not shake off the blues even with help from my family or friends . | 1 | 2 | 3 | 4 |
| 3.I was happy. | 1 | 2 | 3 | 4 |
| 4. I could not get "going." | 1 | 2 | 3 | 4 |
| 5.I enjoyed 1ife. | 1 | 2 | 3 | 4 |
| 6.I fe1t sad. | 1 | 2 | 3 | 4 |
| 7.I felt that every thing I did was an effort. | 1 | 2 | 3 | 4 |
| 8.I felt 1onely . | 1 | 2 | 3 | 4 |
| 9.I had trouble keeping my mind on what I was doing. | 1 | 2 | 3 | 4 |

**3.Basic psychological needs scale**

**Please read each of the following items carefully, thinking about how it relates to your life, and then indicate how true it is for you. Use the following scale to respond:**

| item | Not at all true Very true | | | | | | |
| --- | --- | --- | --- | --- | --- | --- | --- |
| 1. I really like the people I interact with. | 1 | 2 | 3 | 4 | 5 | 6 | 7 |
| 2..Often, I do not feel very competent. | 1 | 2 | 3 | 4 | 5 | 6 | 7 |
| 3. I feel pressured in my life. | 1 | 2 | 3 | 4 | 5 | 6 | 7 |
| 4.People I know tell me I am good at what I do. | 1 | 2 | 3 | 4 | 5 | 6 | 7 |
| 5.I get along with people I come into contact with. | 1 | 2 | 3 | 4 | 5 | 6 | 7 |
| 6.I pretty much keep to myself and don’t have a lot of social contacts. | 1 | 2 | 3 | 4 | 5 | 6 | 7 |
| 7.I generally feel free to express my ideas and opinions. | 1 | 2 | 3 | 4 | 5 | 6 | 7 |
| 8.I have been able to learn interesting new skills recently. | 1 | 2 | 3 | 4 | 5 | 6 | 7 |
| 9.In my daily life, I frequently have to do what I am told. | 1 | 2 | 3 | 4 | 5 | 6 | 7 |
| 10.People in my life care about me. | 1 | 2 | 3 | 4 | 5 | 6 | 7 |
| 11.Most days I feel a sense of accomplishment from what I do. | 1 | 2 | 3 | 4 | 5 | 6 | 7 |
| 12.People I interact with on a daily basis tend to take my feelings into consideration. | 1 | 2 | 3 | 4 | 5 | 6 | 7 |
| 13.In my life I do not get much of a chance to show how capable I am. | 1 | 2 | 3 | 4 | 5 | 6 | 7 |
| 14.There are not many people that I am close to. | 1 | 2 | 3 | 4 | 5 | 6 | 7 |
| 15.I feel like I can pretty much be myself in my daily situations | 1 | 2 | 3 | 4 | 5 | 6 | 7 |
| 16.The people I interact with regularly do not seem to like me much. | 1 | 2 | 3 | 4 | 5 | 6 | 7 |
| 17.I often do not feel very capable. | 1 | 2 | 3 | 4 | 5 | 6 | 7 |
| 18.There is not much opportunity for me to decide for myself how to do things in my daily life. | 1 | 2 | 3 | 4 | 5 | 6 | 7 |
| 19.People are generally pretty friendly towards me. | 1 | 2 | 3 | 4 | 5 | 6 | 7 |

**4.Self-esteem Scale:**

| items | Not at all true Very true | | | | |
| --- | --- | --- | --- | --- | --- |
| 1. I feel that I’m a person of worth, or at least on an equal plane with others | 1 | 2 | 3 | 4 | 5 |
| 2. I feel that I have a number of good qualities | 1 | 2 | 3 | 4 | 5 |
| 3.All in all, I am inclined to feel that I am a failure | 1 | 2 | 3 | 4 | 5 |
| 4.I am able to do things as well as most other people | 1 | 2 | 3 | 4 | 5 |
| 5.I feel I do not have much to be proud of. | 1 | 2 | 3 | 4 | 5 |
| 6.I take a positive attitude toward myself | 1 | 2 | 3 | 4 | 5 |
| 7.On the whole, I am satisfied with myself | 1 | 2 | 3 | 4 | 5 |
| 8.I wish I could have more respect for myself | 1 | 2 | 3 | 4 | 5 |
| 9.I certainly feel useless at times | 1 | 2 | 3 | 4 | 5 |
| 10.I feel that I’m a person of little worth, not on an | 1 | 2 | 3 | 4 | 5 |
